# Validity of verbal autopsy method to determine causes of death among adults in the urban setting of Ethiopia

**DOI:** 10.1186/1471-2288-12-130

**Published:** 2012-08-28

**Authors:** Awoke Misganaw, Damen Haile Mariam, Tekebash Araya, Aderaw Aneneh

**Affiliations:** 1 Addis Ababa Mortality Surveillance Program, College of Health Sciences, Addis Ababa University, Addis Ababa, Ethiopia; 2 College of Health Sciences, Addis Ababa University, Addis Ababa, Ethiopia

**Keywords:** Causes of death, Sensitivity, Specificity, Validity, Ethiopia

## Abstract

**Background:**

Verbal autopsy has been widely used to estimate causes of death in settings with inadequate vital registries, but little is known about its validity. This analysis was part of Addis Ababa Mortality Surveillance Program to examine the validity of verbal autopsy for determining causes of death compared with hospital medical records among adults in the urban setting of Ethiopia.

**Methods:**

This validation study consisted of comparison of verbal autopsy final diagnosis with hospital diagnosis taken as a “gold standard”. In public and private hospitals of Addis Ababa, 20,152 adult deaths (15 years and above) were recorded between 2007 and 2010. With the same period, a verbal autopsy was conducted for 4,776 adult deaths of which, 1,356 were deceased in any of Addis Ababa hospitals. Then, verbal autopsy and hospital data sets were merged using the variables; full name of the deceased, sex, address, age, place and date of death. We calculated sensitivity, specificity and positive predictive values with 95% confidence interval.

**Results:**

After merging, a total of 335 adult deaths were captured. For communicable diseases, the values of sensitivity, specificity and positive predictive values of verbal autopsy diagnosis were 79%, 78% and 68% respectively. For non-communicable diseases, sensitivity of the verbal autopsy diagnoses was 69%, specificity 78% and positive predictive value 79%. Regarding injury, sensitivity of the verbal autopsy diagnoses was 70%, specificity 98% and positive predictive value 83%. Higher sensitivity was achieved for HIV/AIDS and tuberculosis, but lower specificity with relatively more false positives.

**Conclusion:**

These findings may indicate the potential of verbal autopsy to provide cost-effective information to guide policy on communicable and non communicable diseases double burden among adults in Ethiopia. Thus, a well structured verbal autopsy method, followed by qualified physician reviews could be capable of providing reasonable cause specific mortality estimates in Ethiopia. However, the limited generalizability of this study due to the fact that matched verbal autopsy deaths were all in-hospital deaths in an urban center, thus results may not be generalizable to rural home deaths. Such application and refinement of existing verbal autopsy methods holds out the possibility of obtaining replicable, sustainable and internationally comparable mortality statistics of known quality. Similar validation studies need to be undertaken considering the limitation of medical records as “gold standard” since records may not be confirmed using laboratory investigations or medical technologies. The validation studies need to address child and maternal causes of death and possibly all underlying causes of death.

## Background

Many developing countries with the highest burden of poverty and disease continue to lack routine, representative and high quality information on the levels and causes of death [1,2]. Moreover, gross pathology autopsy is neither a practical nor an accepted practice in many developing countries including Ethiopia [3]. This is crucial for health and development policies, health programs; program monitoring and evaluation purposes [4]. In this regard, mortality surveillance systems and demographic surveillance sites (DSS) using validated verbal autopsy procedures suggested being a cost-effective alternative method for ascertaining cause of death and sustainable medium-term solutions [5,6]. As a result, verbal autopsy method has been an epidemiological tool for some decades to estimate cause specific mortality of child, maternal and adult deaths [6,7]. In light of this, for the last few years Ethiopia has established more than six demographic surveillance sites in different regions basing the rural community and an urban based mortality surveillance program that used verbal autopsy to identify causes of death.

The verbal autopsy is interviewing relatives or caregivers about the signs, symptoms, lifestyle behaviors and other characteristics experienced by the deceased before their death and the circumstances surrounding their death [6]. It is based on the assumption that most cause of deaths can be distinguished by their signs and symptoms and that these can be accurately recognized, recalled and reported by lay respondents [8]. In the procedure, several factors may influence the validity and reliability of verbal autopsies; such as the ‘true’ underlying distribution of cause of death in the population, the age and sex of the deceased, quality and standardization of verbal autopsy tool (verbal autopsy questionnaire, diagnostic procedure and mortality classification) and the data collection process [6,9]. As a result, cause of death misclassification can affect the accuracy of the verbal autopsy estimate whenever it creates an imbalance between the number of false positives and the number of false negatives. However, the misclassification by itself doesn’t necessarily indicate the verbal autopsy estimate of cause-specific mortality fraction to be inaccurate [2].

Despite limited validation efforts, verbal autopsy data in Ethiopia is functioning well and become an important source of information to track the growing burden of non communicable diseases, along with the infectious diseases, perinatal and nutritional problems and a base for setting and evaluating health priorities, policies and interventions [10]. Previously, verbal autopsy in Ethiopia has been used to estimate HIV/AIDS and Malaria caused mortality and to evaluate the impact of Anti-Retroviral Therapy (ART) interventions [11,12]. However, to ensure internationally comparable data of known quality, standard verbal autopsy procedures should be promoted and rigorously validated [2,6,13].

For instance, a multicenter validation study in Tanzania, Ethiopia and Ghana indicated the sensitivity and specificity of verbal autopsy for selected adult deaths varied considerably depending on the diagnostic criteria used [9]. Including this, existing few studies all over the world conducted on causes of death in adults have examined few disease conditions and most were focused on children and maternal mortality and infectious diseases [8,9,14-16].

Regardless of its importance, almost no attempt has been made previously to validate verbal autopsy in Ethiopia to see if determined causes closely reflect actual causes of death. These days, there is a growing national concern regarding the validation and performance of verbal autopsy for its sustainability and usefulness in tracking the epidemiological transition [10]. Therefore, the overall purpose of this study is to answer validation questions through measuring sensitivity and specificity of verbal autopsy using hospital medical records as “gold standard”.

## Methods

### Study setting

This validation study was part of Addis Ababa Mortality Surveillance Program (AAMSP) the then project and in place since 2001 in Addis Ababa, Ethiopia. We validated verbal autopsy data with hospital data of the program during 2007 to 2010.

The sampling frame for this verbal autopsy method; the burial surveillance was conducted in all cemeteries (n ≈ 89) of Addis Ababa since 2001. Since cremation is not practiced in Addis Ababa, burials of deaths are conducted at religious or municipality based cemeteries. In principle thus, the burial surveillance captures all deceased residents of Addis Ababa, although biases exist because residents may die and/or be buried outside the capital just as non-residents may be buried inside Addis Ababa. Some of these biases are mostly identified and corrected while others inevitably go unnoticed.

The surveillance registration was conducted by cemetery based clerks who were regularly trained and supervised. Approximately, 20,000 deaths per year were reported by these cemetery clerks. However, due to financial and logistic reasons; randomly 10% of the deaths were drawn from the burial database for verbal autopsy interview.

### Study design

We employed retrospective reviews of burials with verbal autopsy technique and retrospective reviews of hospital records in Addis Ababa, Ethiopia.

### Data collection for verbal autopsy

Three pairs of field workers who are non health professionals who were trained in the technique visited households of the deceased (minimum one month and maximum three months after the funeral) and selected a respondent who was the person most closely associated with the deceased during the terminal illness. The interview was carried out in Amharic (the national language) once its purpose had been fully explained and consent obtained.

Field workers were recruited locally to ensure a common cultural background with the local community. All had completed secondary school, were experienced in conducting surveys, and had demonstrated the ability to conduct a verbal autopsy interview with insight and empathy. The first several interviews of each field worker were carefully monitored and supervised by field coordinators and researchers. Thereafter, weekly feedback sessions were held on a regular basis, providing an opportunity to appraise the quality of information recorded [3]. The completeness of the VA was approximately 85% where refusal accounts nearly 3%, loss of address 6%, unavailability of the care givers with repeated visit 2% and wrong address 4% which may introduce bias in mortality estimations.

The verbal autopsy questionnaire, adapted from a standardized WHO and INDEPTH Network [17,18] was translated into Amharic, back translated into English and modified to reflect culturally recognized accepted terms. The questionnaire was divided into four main parts: an open section where the informant freely describes the symptoms and signs preceding death, and their sequence; followed by a closed section in which a basic filtering question, when answered positively, leads to a more detailed enquiry of the particular symptom. Further sections address identification of the caregivers, use of modern and traditional treatments, and lifestyle practices of the deceased.

The physician review method was used to determine causes of death. Three physicians were participating in the review process which were second or third year internal medicine residents of Addis Ababa University recruited to join the university after serving two or more years as a General Practitioner (GP) in any of the public hospitals. We provided them trainings and annual refreshments on the standard verbal autopsy method.

First, each completed questionnaire was reviewed independently by two physicians. If the same diagnosis was reached, this was accepted as the ‘underlying cause of death’, where not, a third physician made a further blind and independent assessment. If two out of three diagnoses corresponded, this would be accepted; otherwise, the three physicians would set for panel, where consensus achieved would be accepted, if not, the cause of death would be described as ‘undetermined’.

In the review process, almost 10% of the verbal autopsy questionnaires required a third physician review for communicable and non communicable major disease categories, but <1% for injury. Less than 1% of the major disease categories required physicians’ panel. Regarding specific causes of deaths, generally <2% of the verbal autopsy questionnaires required third physicians; and <1% required panel. Finally, the research assistant with health background would assign ICD-10 codes according to the international classification of diseases, 10^th^ revision [19].

Double data entry was done to all the cases, for both the verbal autopsy interview and physician review. Once the data entry was completed, a data manager using STATA driven .do files had been conducting a thorough data cleaning. For the purpose of this paper, we adapted the 2006 Global Burden of Disease classification of causes of death as follows; communicable diseases, non communicable causes, and injuries [20].

### Hospital data collection

A retrospective record review of deaths in 43 public and private hospitals of Addis Ababa from 2007 to 2010 was conducted to validate causes of death reported by verbal autopsy. Nearly, 20,000 adult deaths (15 years and above) of Addis Ababa residents were captured during the study period. Each hospital assigned reviewers who are permanent staffs in the hospitals and centrally three nurses were coordinating, supervising and checking the completeness of the report. Hospital records were assessed by hospital clerks blind to the verbal autopsy diagnosis.

Collected information of validation relevant includes full name, age, sex, and date of death, name of the hospital, and full address of the deceased and the principal cause of death. The data collectors and coordinators had prior relevant experience and provided extensive training on proper review of the medical records and registration books and the use of the data abstraction form.

To capture deaths and complete the relevant information in the hospitals, every attempt was made from patient records and death registry books for patients who died during the study period. In the hospitals diagnosing causes of death was performed by physicians considering patients' history, physical examination, laboratory results and imaging investigations. Only diseases responsible for the death were considered as cause/s of death. Finally, cause of death was coded by nurse coordinators according to the international classification of diseases, 10th revision (ICD-10) [19]. We have listed below specific causes of death with the corresponding ICD-10 assigned.

Cause of Death List: ICD - 10 code; HIV/AIDS: B20–B24; Tuberculosis: A15–A19, B90; Respiratory infections: J00–J06, J10–J18, J20–J22, H65–H66; Meningitis: A39, G00, G03; Malignant neoplasm: C00–C97; Diabetes Mellitus: E10–E14; Cardiovascular diseases: I00–I99; Hypertension: I10–I13; Stroke: I60–I69; Respiratory diseases: J30–J98; Digestive diseases: K20–K92; Chronic Liver Disease: K70, K74; Peptic Ulcer Disease: K25–K27; Genitourinary Diseases: N00–N64, N75–N98; Unintentional injuries: V01–X59, Y40–Y86, Y88, Y89; Road traffic accidents: V01–V04, V06, V09–V80, V87, V89, V99; Intentional injuries (Suicide…): X60–Y09, Y35–Y36, Y870, Y871.

Data were double entered to Access Microsoft office and cleaned using STATA .do files. The 2006 Global Burden of Diseases classification was adapted to classify cause of deaths in our study. This classification categorized diseases into; communicable diseases, non-communicable diseases and all injuries [20].

### Ethical clearance

Verbal Autopsy interview was conducted after obtaining verbal informed consent from the kin or caregiver of the deceased after explaining the purpose and the procedure of the study. Information sheet prepared in English and translated to local language had been provided. Permission for the study had been also obtained from local authorities. Protocol of the program was approved by Institutional Review Board (IRB) of Medical Faculty, Addis Ababa University, and the Ethiopian Science and Technology Agency. Government and institutional officials, religious leaders at each level had been communicated. Individual information was accessible only to the research team and is kept confidential.

### Data management and analysis

The validation consisted of a comparison of verbal autopsy final diagnosis with hospital diagnosis taken as a “gold standard”, followed by calculation of their sensitivity, specificity and positive predictive values. The sensitivity of a verbal autopsy for a particular cause of death such as HIV/AIDS is the proportion of the deceased whose verbal autopsy cause of death is correctly identified as HIV/AIDS out of all those who truly died from HIV/AIDS, while the specificity is the proportion whose cause of death is identified as not HIV/AIDS among those who truly did not die from HIV/AIDS [2].

Verbal autopsy and hospital data sets were merged using the variables; deceased full name, sex, address, age, place of death and date of death. First we found 1356 deaths occurred in hospitals which were reported with verbal autopsy during 2007 to 2010 period. We merged this verbal autopsy data set with hospital data (n = 20,152, age 15 years and above). Finally, we found 335 deaths for this analysis. This was basically due to the incompleteness of the hospital records and registry books; and differences with age, addresses, and deceased full name, place of death or date of death during merging that might introduce bias. The number of causes of death could be greater than the number of deceased adults in the verbal autopsy and hospital diagnosis since we used two or more causes of death (multiple causes of death) and treated independently.

Sensitivity, specificity and positive predictive value analysis was performed for 3 major categories of diseases; communicable diseases, non communicable diseases and injuries and for each of the major diseases under each category such as HIV/AIDS, tuberculosis, malignant neoplasm, cardiovascular diseases etc. We used chi-squared test to compare proportions between selected verbal autopsy adult deaths, selected hospital adult deaths.

Finally, to show the actual causes of death distribution “actual verbal autopsy diagnoses” and for comparison and completeness of this study, cause specific mortality proportion findings of the double mortality burden study included [10]. The current and the former study were from the same study area, data source and study period.

## Results

In public and private hospitals of Addis Ababa, 20,152 adult deaths (15 years and above) were recorded, between October 2007 and 2010. In the same period a verbal autopsy was conducted for 4,776 deaths in Addis Ababa. Of those undergone verbal autopsy, 1,356 were found died in any of Addis Ababa hospitals and the rest were deaths out of hospitals.

### Distribution of cause of death from verbal autopsy and hospital records

The frequency distribution by major disease categories of selected verbal autopsy and hospital deceased adults and burial based actual verbal autopsy diagnosis were almost similar (Table 1). No significant difference was observed. Individual causes of death such as cardiovascular diseases, HIV/AIDS, tuberculosis, digestive diseases and malignancy were also over represented compared to respiratory infections, meningitis, and diabetes and road traffic accidents (Table 2). Except HIV/AIDS, tuberculosis and unintentional injuries, there was no significant difference with cause-specific mortality between selected verbal autopsy adult deaths and actual verbal autopsy diagnosis (Table 2).

**Table 1:** Frequency distribution of causes of death by major categories, verbal autopsy and hospital records

**Cause of Death**	**Selected verbal autopsy deceased adults (n = 335)**		**Selected hospital adult deaths (n = 335)**		**Actual verbal autopsy diagnoses **[10]
	**Number**	**% (95% CI)**	**Number**	**% (95% CI)**	**% (95% CI)**
Communicable Diseases	145	43(38–49)	124	37 (32–42)	42 (40.6–43.8)
Non Communicable Diseases	159	48 (42–53)	181	54 (49–59)	51 (49.7–52.9)
Injuries	23	7(4–10)	27	8 (5–11)	6 (5.5–7.0)
Undetermined	10	3 (1–5)	3	1	7 (6.0–7.7)
**Total**	335*	**100**	**335**	**100**	100*

^*^ VA used multiple causes of death diagnoses and may not add up exactly and give 335 or 100%.

**Table 2:** Frequency distribution of causes of death from VA and hospital records

**Cause of Death**	Selected VA deceased adults (n = 335)*		Selected hospital adult deaths (n = 335)*		**Actual VA diagnoses **[10]
	**Number**	**% ( 95% CI)**	**Number**	**% (95% CI)**	**% (95% CI)**
Communicable Diseases					
HIV/AIDS	97	29 (24–34)	69	21(16–25)	19 (17.8–20.3)
Tuberculosis	77	23 (18–28)	48	14 (11–18)	12 (11.3–13.5)
Respiratory Tract Infections	14	4 (2–6)	14	4 (2–6)	3 (2.2–3.3)
Meningitis	7	2 (0.5–4)	7	2 (0.5–4)	1
Non Communicable Diseases					
Malignant neoplasm	28	8 (5–11)	28	8 (5–11)	10 (9.4–11.3)
Diabetes Mellitus	15	4 (2–7)	14	4 (2–7)	5 (4.2–5.6)
Cardiovascular diseases	75	22 (18–27)	83	25 (20–29)	24 (22.5–25.2)
Hypertension	38	11(8–15)	22	7 (4–9)	12 (10.5–12.5)
Stroke	33	10 (7–13)	25	7 (5–10)	11(9.9–11.9)
Respiratory diseases	7	2 (0.5–4)	17	5 (3–7)	3 (2.5–3.6)
Digestive Diseases	34	10 (7–13)	36	11(7–14)	9 (7.9–9.7)
Chronic Liver Disease	17	5 (3–7)	12	4 (2–6)	4 (3.8–5.1)
Peptic Ulcer Disease	2	1	5	2(1–4)	1
Genitourinary Diseases	12	4 (2–6)	8	2 (1–4)	4 (3.1–4.3)
Injuries					
Unintentional	21	6 (4–9)	14	4 (2–6)	3 (2.2–3.3)
Road traffic accidents	17	4(3–7)	14	2(2–6)	2
Intentional (Suicide…)	2	1	2	1	2
Undetermined	10	3 (1–5)	2	1	7 (6.0–7.7)
Total	335	100	335	100	100

*Total may not be exactly 335 because multiple causes of death used in the verbal autopsy and hospital diagnosis.

### Validity of verbal autopsy diagnosis by hospital diagnosis

Table 3 shows the sensitivity, specificity and positive predictive value for three major category causes of death; communicable diseases, non communicable diseases and injury. Among the communicable disease category, we were able to show four causes of death (i.e. HIV/AIDS, tuberculosis, respiratory tract infection and meningitis). Likewise, from the non communicable disease category; we were able to calculate the above values for six sub category causes of death (i.e. malignant neoplasm, diabetes, cardiovascular diseases, respiratory diseases, digestive diseases and genitourinary diseases) and for three individual causes of death (hypertensive heart diseases, stroke and chronic liver disease). The sensitivity, specificity and positive predictive value of unintentional injuries with road traffic accident were also calculated (Table 3).

**Table 3:** Validity of VA diagnosis by hospital diagnosis

**Cause of death**	**Sensitivity (%,95% CI)**	**Specificity (%,95% CI)**	**PPV (%,95%CI)**
**Communicable Diseases category**	79 (72–86)	78 (72–83)	68 (60–76)
HIV/AIDS	68 (57–79)	81 (76–86)	50 (40–60)
Tuberculosis	63 (49–76)	84 (79–88)	39 (28–50)
Respiratory Tract Infections	14 (5–33)	96 (94–98)	14 (4–32)
Meningitis	71 (35–100)	99 (98–100)	71 (37–100)
**Non Communicable Disease**	69 (62–76)	78 (71–85)	79 (73–85)
Malignant neoplasm	46 (28–65)	95 (93–98)	46 (28–64)
Diabetes Mellitus	57 (30–84)	98 (96–99)	53 (28–78)
Cardiovascular diseases	65 (55–75)	91 (88–95)	72 (62–82)
Hypertension	59 (34–80)	92 (89–95)	34 (19–49)
Stroke	68 (49–87)	95 (92–97)	52 (35–69)
Respiratory diseases	24 (3–44)	99 (98–100)	57 (20–93)
Digestive Diseases	44 (28–61)	94 (91–97)	47 (30–64)
Chronic Liver Disease	67 (39–94)	97 (95–99)	47 (23–71)
Genitourinary Disease	38 (2–73)	97 (95–99)	25 (0.5–50)
**Injury**	70 (53–88)	98 (97–100)	83 (68–98)
Unintentional	86 (67–100)	97 (95–99)	57 (36–78)
Road traffic accidents	86 (67–100)	98 (97–100)	71 (49–93)

### Communicable diseases

Among 124 hospital adult deaths with communicable disease, sensitivity of the verbal autopsy diagnoses was 79% (CI, 72%–86%), specificity 78% (CI, 72%–83%) and positive predictive value 68% (CI, 60%–76%). High sensitivity was achieved for HIV/AIDS 68% (CI, 57%–79%), tuberculosis 63% (CI, 49%–76%), and meningitis 71% (CI, 35%–100%, although numbers were small for meningitis category, *n* = 7) (Tables 2 and 3). Specificity values for these diseases were above 80% and reached 99%.

### Non-communicable diseases

For the 181 cases of non-communicable diseases, sensitivity of the verbal autopsy diagnoses was 69% (CI, 62%–76%), specificity 78% (CI, 71%–85%) and positive predictive value 79% (CI, 73%–85%). Sensitivity was highest for stroke 68% (CI, 49%–87%), and lower for respiratory diseases 24% (CI, 3%–44%). Sensitivity values of malignant neoplasm 46%, diabetes 57%, cardiovascular disease 63%, hypertension 67%, chronic liver disease 44%, digestive diseases 59% and genitourinary diseases 38% were reported in Table 3.

### Injuries

For the 27 hospital cases of injury adult deaths, sensitivity of the verbal autopsy diagnoses was 70% (CI, 53%-88%), specificity 98% (CI, 97%-100%) and positive predictive value 83% (CI, 68%-98%). Sensitivity of the road traffic accident was 86% (CI, 67%-100%) and specificity 98% (CI, 97%-100%). Intentional injury deaths such as suicide and homicide; however the numbers were too small to validate separately (Table 3).

### Cause of death misclassification in verbal autopsy

Table 4 shows the effect of misclassification of causes of death in verbal autopsy validity. Sensitivity, specificity, false positives and false negatives of verbal autopsy differed between causes of death (Table 3). About 47 and 50 adult deaths respectively were tuberculosis and HIV/AIDS false positives; 18 and 22 adult deaths respectively were also tuberculosis and HIV/AIDS false negatives. False positives of 21 adult deaths and false negatives of 29 adult deaths were observed among cardiovascular disease categories (Table 4).

**Table 4:** Causes of death misclassification in VA

VA Diagnosis *	Hospital Diagnosis “gold standard“ *											**Total**
	**AIDS**	**TB**	**RTI**	**Meningitis**	**Malignant**	**DM**	**CVD**	**RD**	**DD**	**GD**	**Unintentional Injury**	
AIDS	**47**	5	6	2	4	1	11	3	5	1	0	**97**
TB	5	**30**	4	1	2	0	12	3	4	0	0	**77**
RTI	2	2	**2**	0	1	0	2	1	1	0	0	**14**
Meningitis	1	0	0	**5**	0	0	0	0	0	0	0	**7**
Malignant	4	4	1	0	**13**	0	4	0	0	0	1	**28**
DM	2	1	0	0	0	8	2	0	1	1	0	**15**
CVD	5	0	2	0	3	2	54	4	3	3	0	**75**
RD	0	0	0	0	1	0	1	4	1	0	0	**7**
DD	3	4	0	0	1	1	2	0	16	1	0	**34**
G D	2	3	1	0	1	0	2	1	0	3	0	**12**
Unintentional Injury	0	0	0	0	0	0	1	0	1	0	12	**21**
**Total**	**69**	**48**	**14**	**7**	**28**	**14**	**83**	**17**	**36**	**8**	**13**	**335**

* Total may not be exactly 335 because multiple causes of death used in the verbal autopsy and hospital diagnosis.

AIDS = HIV/AIDS, TB = Tuberculosis, RTI = Reparatory Tract Infection, DM = Diabetes Miletus, CVD = cardiovascular disease, RD = Respiratory Disease, DD = Digestive Diseases, GD = Genitourinary Diseases.

## Discussion

We conducted this validation study in the urban setting of Ethiopia, where double burden of communicable and non communicable diseases observed. We used verbal autopsy data between 2006 and 2009 to identify the double burden in mortality from communicable diseases (42%) and non communicable diseases (51%) with statistically significant difference [10]. The big concern with these finding was lack of validation study from Ethiopian context. To extend the benefits our findings to health policy makers we recognized the need to validate our verbal autopsy method [21].

In our validation study, the cause specific mortality proportions of selected hospital diagnosed deaths; communicable diseases, non communicable diseases and injuries were comparable with the actual verbal autopsy burial based study. These parallel results may indicate the mortality burden in the population has been reflected in the hospitals; though hospital data lack representativeness to the bigger population. Such cause-specific mortality statistics remain scarce for the majority of low income countries where the highest disease burdens are experienced. The expansion of mortality surveillance programs and DSS sites with verbal autopsy method complemented with hospital data represents the most promising interim solution in countries lacking adequate or representative data [21].

To monitor the epidemiological transition in Ethiopia; an urban based mortality surveillance program and six rural based DSS sites were established during the last 5 years. In all these sites, verbal autopsy data are regularly available to identify causes of death. Although, verbal autopsy process has several stages and many factors can influence the level of its accuracy [22]; almost none of the above sites did validation study to show its significance and maximize the utility of verbal autopsy data. In relation to this, identifying the degree of uncertainty with verbal autopsy method is critical while utilizing the verbal autopsy information at different levels of health decision makings, which will vary by causes of death [21]. In this sense, our validation study is of paramount important to make a verbal autopsy method a crude substitute for medical certification of several causes of death from Ethiopian context.

Validation study of verbal autopsy faces the question of how to obtain a suitable reference diagnosis. Several studies have used causes of death based on medical records as the “gold standard” [4,9,14-16,23]. However, validation studies should also take into consideration the limitation of medical records of diagnosis as “gold standard”. Physician diagnosis of medical records may or may not be supported by different diagnostic tests, which may affect the accuracy of the “gold standard”. In countries where different diagnostic tests are lacking and the health information system relies on health facility reports; we believe that verbal autopsy would be a useful tool if the verbal autopsy diagnoses correlated well with hospital diagnosis; in other words, if verbal autopsy gave information as good as the hospital physicians are currently providing [9].

Studies indicated that validation of verbal autopsy is considered to have an acceptable level of diagnostic accuracy at the population level, if sensitivity is at least 50% and specificity at least 90% [24]. Our finding of verbal autopsy in detecting causes of death by major categories is by far larger than the acceptable level of sensitivity of 69% to 79% and specificity of injury 98%, but specificity is lower for communicable and non communicable diseases 78%.

Of the leading causes of death in the actual verbal autopsy study, cardiovascular diseases, HIV/AIDS, tuberculosis and diabetes were identified with sensitivity larger than the acceptable level. The specificity was higher for cardiovascular diseases and diabetes, but slightly lower for HIV/AIDS and tuberculosis compared with the level. Respiratory infectious diseases, malignant neoplasm, chronic respiratory disease, digestive diseases and genitourinary diseases were identified with sensitivity below the level.

Individual diseases such as meningitis, hypertension, stroke, and chronic liver disease and road traffic accidents were also identified with sensitivity larger than the acceptable level. Regarding specificity, except HIV/AIDS and Tuberculosis all were identified above the acceptable level.

On top of this, the above criteria of validation (sensitivity at least 50% and specificity at least 90%) is not uniformly regarded as acceptable [25] because low sensitivity and specificity does not necessary imply low level of accuracy; or relatively high sensitivity and specificity may result serious misclassification errors. In the case of low sensitivity and specificity, the false positives and false negatives may counterbalance, and may not affect the verbal autopsy accuracy [2,9].

The specificity of verbal autopsy was found to be highest for the groups of injury category compared to communicable and non communicable categories. This is in line with a multicenter study finding from Ethiopia, Ghana and Tanzania with specificity (97%) of the injuries. Similarly the communicable disease categories’, sensitivity (82%) and specificity (78%); and the non communicable disease categories’ sensitivity (71%) and specificity (87%) were very similar to ours findings [9]. Comparing with the above study, our sensitivity finding of HIV/AIDS, tuberculosis, cardiovascular diseases and neoplasm was higher. Except HIV/AIDS and tuberculosis, the specificity of the above mentioned causes of death were almost similar [9].

The effect of misclassification of causes of death in verbal autopsy was more observed with HIV/AIDS and tuberculosis compared with other causes. In both cases false positives were higher than false negatives implies overestimating the results. The possible explanation could be, HIV/AIDS and tuberculosis are priority public health problems in the health system with a better diagnostic facility and resources allocation. On the other hand, there could be a bias with our surveillance program; initially was funded aiming HIV/AIDS related mortality. Physicians subjectivity during review could also introduce bias, however the published levels of inter-observer reliability are generally high but may merely reflect the expectation of the individual reviewers, who are aware of the epidemiological pattern and characteristics of diseases in their area [24].

Misclassification affects the accuracy of the verbal autopsy estimate whenever it creates an imbalance between the number of false positives and false negatives [2]. When there is an excess of false positive over false negatives on HIV/AIDS and tuberculosis, the estimate of cause specific mortality fraction based on verbal autopsy is an overestimate. Conversely, when there is an excess of false negatives over false positives on cardiovascular diseases, it is an underestimate. When the number of false positives equals the number of false negatives, the errors would counterbalance and therefore they do not affect the verbal autopsy estimate [2].

## Conclusions

Although the cause structure of our validation sample resembles that of the general population to which the results are to be applied, our findings from selected hospital deaths might have limitation of generalizability. Our validation samples were also the deceased who died in the hospitals. The response to verbal autopsy questions might have been influenced by the response of caregivers and family members who got information from health professionals compared to the deceased at home. Of course, we do not have any practical alternative to carrying out this verbal autopsy validation study without these respondents (whose causes of death knowledge might have been influenced by contacts with health workers).

With the above limitations, our validation study indicates the importance of verbal autopsy as a source of data for population lacking other reliable sources of mortality information. This study indicates that verbal autopsy has the potential to provide cost-effective information to guide policy, set priorities and track impacts. A well structured verbal autopsy tool, followed by qualified physician reviews is capable of providing a reasonable confirmation on mortality estimates. Such application and refinement of existing verbal autopsy method holds out the possibility of obtaining replicable, sustainable and internationally comparable mortality statistics of known quality in Ethiopia. Thus, similar validation studies need to be undertaken considering the limitation of medical records as “gold standard”.

## Competing interests

The author(s) declare that they have no competing interests.

## Authors’ contributions

AM: he has made substantial contributions to conception and design, acquisition of data, analysis and interpretation of data, draft the manuscript and revising it critically for important intellectual content. DHM: he has made substantial contributions to conception and design, drafting the manuscript or revising it critically for important intellectual content and given final approval of the version to be published. TA: she has made substantial contributions to conception and design and involved in drafting the manuscript. AA: has made substantial contributions to conception and design and involved in drafting the manuscript. All authors read and approved the final manuscript.

## Authors’ information

Awoke Misganaw is a public health researcher in Addis Ababa Mortality Surveillance Program, College of health sciences, Addis Ababa University. He is a PhD candidate in public health, in the School of Public Health, Addis Ababa University. He has earned his Masters Degree in Public Health, School of Public Health, Addis Ababa University, Ethiopia.

Damen Haile Mariam is a public health professor in Addis Ababa University. Currently, he is senior researcher and Post Graduate and Research Associate Dean for College of Health Sciences, Addis Ababa University, Ethiopia

Tekebash Araya is Addis Ababa Mortality Surveillance Program Manager, College of health sciences, Addis Ababa University. She is a PhD candidate in public health, in the School of Public Health, Addis Ababa University. She has earned her Masters Degree in Public Health, from School of Public Health, Addis Ababa University, Ethiopia.

Aderaw Anteneh is a demographer researcher in Addis Ababa Mortality Surveillance Program, College of health sciences, Addis Ababa University. He has earned his Masters Degree in Demography, Population Studies, Addis Ababa University, Ethiopia.

## Pre-publication history

The pre-publication history for this paper can be accessed here:

http://www.biomedcentral.com/1471-2288/12/130/prepub
